# Urologic chronic pelvic pain syndrome 3‐year symptom trajectories: the Multidisciplinary Approach to the Study of Chronic Pelvic Pain (MAPP) Symptom Patterns Study

**DOI:** 10.1111/bju.70087

**Published:** 2025-12-02

**Authors:** Catherine S. Bradley, Mengying You, Wensheng Guo, Niloofar Afari, Priyanka Gupta, Karl J. Kreder, John N. Krieger, H. Henry Lai, Susan K. Lutgendorf, Bruce D. Naliboff, Siobhan Sutcliffe, Frank Tu, David A. Williams, Tara McWilliams, Larissa Rodriguez, J. Richard Landis, for the Multidisciplinary Approach to the Study of Chronic Pelvic Pain (MAPP) Research Network

**Affiliations:** ^1^ Department of Obstetrics and Gynecology, Carver College of Medicine University of Iowa Iowa City IA USA; ^2^ Department Urology, Carver College of Medicine University of Iowa Iowa City IA USA; ^3^ Department of Psychological and Brain Sciences, Obstetrics and Gynecology and Urology University of Iowa Iowa City IA USA; ^4^ Department of Biostatistics, Epidemiology and Informatics, Perelman School of Medicine University of Pennsylvania Philadelphia PA USA; ^5^ Department of Psychiatry University of California San Diego School of Medicine San Diego CA USA; ^6^ Department of Medicine David Geffen School of Medicine at UCLA Los Angeles CA USA; ^7^ Department of Urology University of Michigan Ann Arbor MI USA; ^8^ Department of Anesthesiology University of Michigan Ann Arbor MI USA; ^9^ Department of Urology University of Washington School of Medicine Seattle WA USA; ^10^ Department of Surgery (Urology) and Anesthesiology Washington University School of Medicine St Louis MO USA; ^11^ Division of Public Health Sciences, Department of Surgery Washington University School of Medicine St. Louis MO USA; ^12^ Endeavor Health and Pritzker School of Medicine University of Chicago Chicago IL USA; ^13^ Department of Urology Weill Cornell Medicine New York NY USA; ^14^ School of Statistics and Data Science Shanghai University of International Business and Economics Shanghai China

**Keywords:** interstitial cystitis, bladder pain syndrome, chronic prostatitis, cohort study, epidemiology

## Abstract

**Objectives:**

To characterise 3‐year pelvic pain and urinary symptom trajectories and to identify baseline factors associated with urologic chronic pelvic pain syndrome (UCPPS) improvement.

**Patients and Methods:**

The Trans‐Multidisciplinary Approach to the Study of Chronic Pelvic Pain (MAPP) Symptom Patterns Study was a multicentre, prospective cohort study of UCPPS, including interstitial cystitis/bladder pain syndrome and chronic prostatitis/chronic pelvic pain syndrome. Patients completed four weekly run‐in assessments, baseline visit, and quarterly visits up to 3 years, providing clinical and patient‐reported data. A functional clustering approach, applied separately to Pelvic Pain Severity (PPS) and Urinary Symptom Severity (USS) longitudinal change scores, was used to generate symptom trajectory clusters dichotomised as Group 0 ‘improvers’ vs Groups 1–3 ‘non‐improvers’. Logistic regression models explored baseline factors associated with improvement and included run‐in period average and baseline scores to adjust for regression to the mean effects.

**Results:**

A total of 545 patients (66% female) were followed for a median (interquartile range) of 34 (23–35) months. Four trajectory clusters were identified for each of PPS and USS, consistent with moderate improvement (Group 0), slight improvement (Group 1), no change (Group 2), and slight worsening (Group 3). In all, 18% and 19% of patients were in the moderately improved PPS and USS groups, respectively, representing 30% of patients overall. Female sex, better sleep, and less opioid use were associated with PPS improvement (Group 0); younger age and baseline cystoscopic treatment were associated with USS improvement (Group 0).

**Conclusion:**

In all, 30% of patients with UCPPS demonstrated improvement in pain and/or urinary symptoms over 3 years. Baseline factors associated with improvement may represent markers of a milder or localised phenotype and/or treatment effects.

**Abbreviations**
CP/CPPS: chronic prostatitis/chronic pelvic pain syndrome
EPS: Epidemiology and Phenotyping Study
GRA: Global Responses Assessment
GUPI: Genitourinary Pain Index
IC/BPS: **i**nterstitial cystitis/bladder pain syndrome
ICSI: Interstitial Cystitis Symptom Index
IQR: interquartile range
MAPP: Multidisciplinary Approach to the Study of Chronic Pelvic Pain
OR: odds ratio
PPS: Pelvic Pain Severity
PROMIS: Patient‐Reported Outcomes Measurement Information System
QOL: quality of life
SF‐12: 12‐item Short‐Form Health Survey
SPS: Symptom Patterns Study
UCPPS: urologic chronic pelvic pain syndrome
USS: Urinary Symptom Severity

## Introduction

Urologic chronic pelvic pain syndrome (UCPPS), including interstitial cystitis/bladder pain syndrome (IC/BPS) and chronic prostatitis/chronic pelvic pain syndrome (CP/CPPS), is a debilitating condition. Despite increasing research, the long‐term natural history of UCPPS is not well described, in part because prior research has studied inconsistent disease definitions and populations.

Previous Multidisciplinary Approach to the Study of Chronic Pelvic Pain (MAPP) Research Network studies have provided insights using standardised outcomes and assessment. The 1‐year Trans‐MAPP Epidemiology and Phenotyping Study (EPS) provided shorter‐term assessment of UCPPS symptom trajectories [1]. A ‘functional clustering’ approach was developed to discover and validate subgroups with improving, stable, and worsening symptoms over the 1‐year period. Predictors of worsening symptoms included widespread pain, non‐urological symptoms, and poorer physical health. Research in a subset of the same MAPP EPS patients, followed for ≥8 years, found little additional change occurred, on average, in pain and urinary symptoms [2].

The Trans‐MAPP Symptom Patterns Study (SPS), a larger 3‐year observational study, was designed to further investigate clinical factors associated with longitudinal symptom changes and treatment responses [3]. Our objectives were to identify 3‐year pelvic pain and urinary symptom trajectories using a similar functional clustering approach in a cohort of well‐characterised patients with UCPPS and to describe baseline predictors associated with these trajectory groups. We hypothesised that we would identify UCPPS patient subgroups with meaningful symptom change.

## Patients and Methods

### Study Design/Patients

The Trans‐MAPP SPS was a prospective, observational study of UCPPS over a 3‐year follow‐up [3]. Six sites and a data coordinating centre participated. The study was approved by Institutional Review Boards at each site and was performed according to the Declaration of Helsinki, and patients signed informed consents. Patients were enrolled between July 2015 and February 2019. Follow‐up visits were performed for up to 3 years and/or until June 2020, when data collection ended. The Strengthening the Reporting of Observational Studies in Epidemiology (STROBE) statement was followed in preparing this report [4].

Adult women and men with clinical diagnoses of IC/BPS or CP/CPPS who reported pelvic pain of ≥1 (0–10 scale) associated with urinary symptoms for ≥3 months were enrolled. Exclusion criteria included lower urinary tract pathology or conditions whose symptoms might overlap with UCPPS (e.g., neurological disorder affecting the bladder and pelvic cancer; see Supporting Information for detailed eligibility criteria). Patients completed a screening visit (Week 0), three run‐in assessments (Weeks 1–3; on‐line), and a baseline visit at Week 4 [3]. Follow‐up included quarterly on‐line assessments and in‐person visits at 6, 18, and 36 months. Patients with at least one visit beyond baseline were included.

### Baseline Measures

Baseline data included demographics, medical history, examination, and treatments (within current month). UCPPS treatments were categorised by type and mechanism of action (Table 1).

**Table 1:** Baseline treatments reported in the SPS and ordinal treatment mechanism categories in the 545 patients.

UCPPS treatment (active within last month)	*N* (%)	SPS ordinal treatment mechanism category*
		0 = None
		1 = Peripheral
		2 = Central
		3 = Opioid
**Medications**		
Alpha adrenergic blocker	76 (13.9)	1
Pentosan polysulfate	116 (21.3)	1
Neuropathic pain medication	31 (5.7)	2
Tricyclic antidepressant	141 (25.9)	2
Oral opioid	127 (23.3)	3
Other	–	0, 1, 2
**Non‐medicine treatments**		
Bladder instillations (home)	12 (2.2)	1
Bladder instillations (office)	50 (9.2)	1
Bladder training	77 (14.1)	1
Dietary changes	323 (59.3)	1
Heat/cold application	231 (42.2)	1
Pelvic floor exercises	100 (18.3)	1
Pelvic floor physical therapy	74 (13.6)	1
Acupuncture	22 (4.0)	2
Counselling/psychotherapy	48 (8.8)	2
Home exercise/yoga	183 (33.6)	2
Massage	41 (7.5)	2
**Procedures/surgery**		
Intradetrusor botulinum toxin injection	1 (0.2)	1
Cystoscopic treatment (including hydrodistension and Hunner's ulcer treatment)	19 (3.5)	1
Sacroneuromodulation	20 (3.7)	1

* Treatments for UCPPS were categorised into groups by likely mechanism of action for UCPPS and type, including none (0), peripheral action (1), central action (2), and oral opioids (3) for medications; and peripheral (1) and central (2) action for non‐medicine treatments.

The SPS patient‐reported assessments are listed in Table S1 and summarised here. The Genitourinary Pain Index (GUPI) [5] and Interstitial Cystitis Symptom Index (ICSI) [6] were used to assess UCPPS pain, urinary symptoms, and quality of life (QOL). Non‐urological pain measures assessing widespread pain included the Brief Pain Inventory and MAPP II body map, among others [7, 8]. Additional questionnaires assessed health‐related QOL (12‐item Short‐Form Health Survey [SF‐12] [9]) and sleep quality (Patient‐Reported Outcomes Measurement Information System [PROMIS] Fatigue and Sleep Disturbance [10]), as well as overlapping pain conditions, depression, anxiety, and stress. Patients were also asked if they were currently experiencing a flare of their urological or pelvic pain symptoms (‘symptoms that are much worse than usual’) or experienced a flare within the past 3 months. Last, a Global Responses Assessment (GRA) measured patient‐reported improvement or worsening: ‘As compared to when you started the study, how would you rate your overall symptoms now?’ with responses ‘markedly worse’, ‘moderately worse’, ‘slightly worse’, ‘no change’, ‘slightly improved’, ‘moderately improved’, or ‘markedly improved’. Responses of at least moderately improved were considered improvement vs all others (not improved). Experimental pain sensitivity was measured using pressure pain testing at the right thumb as previously described [3, 11].

### Longitudinal Symptom Trajectory Patterns Using Functional Clustering Approach

Pelvic Pain Severity (PPS: range 0–28) and Urinary Symptom Severity (USS: range 0–25) composite scores, created from the GUPI and ICSI items and included in several MAPP analyses [2, 12, 13, 14], were used to generate separate longitudinal trajectories for pain and urinary symptom change. A score change of 4 (PPS) or 3 (USS) has been shown to represent clinically important differences based on 3–6 month follow‐up data [15]. However, in order to incorporate non‐linear, highly variable within‐person change profiles over a longer 3‐year timeframe, a functional clustering approach [16] was used to cluster the patients into subgroups based on their longitudinal trajectory patterns separately for each of the two primary UCPPS symptom severity scores (PPS, USS). Our algorithm uses a cross‐validation Kullback–Leibler divergence criterion to choose the optimal number of groups resulting in four subgroups for each outcome, ranging from ‘moderate improvement’, ‘slight improvement’, ‘no change’, and ‘slight worsening’ [16]. As the primary focus of our study is to identify baseline factors that are predicting symptom improvement, we create a binary outcome by combining ‘slight improvement’, ‘no change’, and ‘slight worsening’ into one group in comparison with ‘moderate improvement’. Our functional clustering algorithm was implemented in MATLAB (MathWorks, 2021a; Natick, MA, USA), clustering on the individual longitudinal change scores. The statistical methods for this approach were previously reported and are described in further detail in Supporting Information [1, 14, 16]. To enable the calculation of within‐patient longitudinal change, missing values at the baseline in‐clinic visit (4 weeks) were imputed using a single imputation procedure based on a linear mixed‐effects model (see Supporting Information).

### Run‐in Period Averages

To better account for week‐to‐week symptom variability within‐person and to improve adjustments for regression to the mean, average scores during the four weekly run‐in visits were calculated for primary UCPPS symptom measures, including PPS and USS scores, as well as for selected covariates. For a binary symptom (present [1] or absent [0]) at each run‐in period contact, this run‐in period average captures the propensity (ranging from 0.0 and 1.0) for repeated measures of a specific binary symptom to be present. These average scores derived from the run‐in period were then included as candidate covariates, together with baseline measures, separately for PPS and USS symptom trajectory outcome models. A wide array of baseline and run‐in variables was considered for inclusion in the symptom trajectory models, including demographics, overlapping pain conditions, widespread pain scores via body maps, current treatments, UCPPS symptom and QOL assessments (individual items and scores), QOL, lifestyle, and psychosocial measure scores, and pressure–pain thresholds (see Table 2 and Table S2).

**Table 2:** Baseline patients’ characteristics overall and by PPS and USS trajectories (cluster 0 [moderately improved] vs clusters 1–3 [all others]) (*n* = 545).

Characteristic	Level	*N*	Overall	PPS: 0	PPS: 1–3	*P*	USS: 0	USS: 1–3	*P*
			*N* = 545	*N* = 98	*N* = 447		*N* = 102	*N* = 443	
Age, years, mean (sd)		545	45.48 (15.61)	46.02 (16.81)	45.36 (15.36)	0.81	40.95 (14.24)	46.52 (15.75)	**0.001**
Gender, *n* (%)	Female	545	361 (66.24)	76 (77.55)	285 (63.76%)	**0.009**	80 (78.43)	281 (63.43)	**0.004**
	Male		184 (33.76)	22 (22.45)	162 (36.24%)		22 (21.57)	162 (36.57)	
Race, *n* (%)	White	545	497 (91.19)	91 (92.86)	406 (90.83%)	0.76	91 (89.22)	406 (91.65)	0.58
	Black		31 (5.69)	5 (5.10)	26 (5.82%)		8 (7.84)	23 (5.19)	
	Other		17 (3.12)	2 (2.04)	15 (3.36%)		3 (2.94)	14 (3.16)	
Education, *n* (%)	< College degree	545	171 (31.38)	32 (32.65)	139 (31.10%)	0.078	34 (33.33)	137 (30.93)	0.84
	College degree		238 (43.67)	50 (51.02)	188 (42.06%)		42 (41.18)	196 (44.24)	
	Grad/prof degree		136 (24.95)	16 (16.33)	120 (26.85%)		26 (25.49)	110 (24.83)	
Annual income, *n* (%)	≥$50K	494	181 (36.64)	30 (35.71)	151 (36.83%)	0.79	34 (36.56)	147 (36.66)	0.18
	>$50K to $100K		173 (35.02)	32 (38.10)	141 (34.39%)		39 (41.94)	134 (33.42)	
	>$100K		140 (28.34)	22 (26.19)	118 (28.78%)		20 (21.51)	120 (29.93)	
Symptom duration, years, mean (sd)		539	12.26 (11.75)	12.09 (11.89)	12.29 (11.73)	0.90	10.48 (10.96)	12.66 (11.90)	0.059
Body mass index, kg/m^2^, mean (sd)		544	27.24 (5.76)	26.75 (5.09)	27.34 (5.89)	0.36	27.01 (6.51)	27.29 (5.58)	0.090
COPC, *n* (%)		488	312 (63.93)	60 (65.93)	252 (63.48%)	0.66	70 (76.92)	242 (60.96)	**0.004**
No. of non‐pelvic body map regions with pain, *n* (%)	None (0)	535	123 (22.99)	26 (26.80)	97 (22.15%)	0.26	24 (23.76)	99 (22.81)	0.90
	Intermediate (1–2)		244 (45.61)	37 (38.14)	207 (47.26%)		44 (43.56)	200 (46.08)	
	Widespread (3–7)		168 (31.40)	34 (35.05)	134 (30.59%)		33 (32.67)	135 (31.11)	
History of Hunner's lesion on cystoscopy, *n* (%)	No	545	181 (33.2)	36 (36.7)	145 (32.4%)	0.22	38 (37.3)	143 (32.3)	0.14
	Yes		28 (5.1)	9 (9.2)	19 (4.3%)		10 (9.8)	18 (4.1)	
	Missing		336 (61.7)	53 (54.1)	283 (63.3%)		54 (52.9)	282 (63.7)	
Cystoscopic UCPPS treatment within past month, *n* (%)		543	19 (3.5)	5 (5.2)	14 (3.1%)	0.33	7 (6.9)	12 (2.7)	**0.038**
Medication taken for UCPPS pain (category)^†^, *n* (%)	0: None	545	90 (16.5)	23 (23.5)	67 (15.0%)	0.13	22 (21.6)	68 (15.4)	0.41
	1: Peripheral		187 (34.3)	35 (35.7)	152 (34.0%)		32 (31.4)	155 (35.0)	
	2: Central		141 (25.9)	23 (23.5)	118 (26.4%)		23 (22.6)	118 (26.6)	
	3: Opioid		127 (23.3)	17 (17.4)	110 (24.6%)		25 (24.5)	102 (23.0)	
Medication taken for UCPPS pain (ordinal category 0–3)*, mean (sd)		545	1.56 (1.02)	1.35 (1.03)	1.61 (1.02)	**0.023**	1.50 (1.09)	1.57 (1.01)	0.51
No current UCPPS treatment, *n* (%)		545	27 (5.0)	6 (6.1)	21 (4.7)	0.56	9 (8.8)	18 (4.1)	**0**.**046**
Current flare, *n* (%)		543	109 (20.1)	34 (35.1%)	75 (16.8)	**<0.001**	28 (27.5)	81 (18.4)	**0.039**
Flare in last 3 months, *n* (%)		542	421 (77.7)	82 (84.5%)	339 (76.2)	0.073	87 (85.3)	334 (75.9)	**0.040**
Non‐PPS (0–10), mean (sd)		543	3.46 (2.65)	3.44 (2.56)	3.46 (2.67)	0.95	3.72 (2.84)	3.40 (2.60)	0.39
SF‐12 physical component score, mean (sd)		542	45.54 (10.01)	46.31 (9.03)	45.38 (10.22)	0.60	44.32 (10.16)	45.82 (9.97)	0.17
SF‐12 mental component score, mean (sd)		541	43.50 (10.71)	42.55 (11.25)	43.70 (10.59)	0.52	41.46 (11.15)	43.96 (10.57)	0.056
PROMIS fatigue, mean (sd)		536	54.93 (10.00)	55.79 (9.34)	54.74 (10.14)	0.22	57.23 (10.79)	54.39 (9.75)	**0.010**
PROMIS sleep disturbance, mean (sd)		537	54.53 (9.56)	53.85 (9.47)	54.68 (9.58)	0.42	55.82 (9.92)	54.23 (9.46)	0.090
HADS depression, mean (sd)		535	5.68 (4.55)	5.89 (4.50)	5.63 (4.57)	0.52	6.33 (4.70)	5.53 (4.51)	0.11
HADS anxiety, mean (sd)		533	7.31 (4.82)	7.62 (5.03)	7.24 (4.78)	0.57	8.59 (5.29)	7.02 (4.66)	**0.007**
Perceived stress scale, mean (sd)		532	15.80 (8.22)	17.20 (7.64)	15.49 (8.31)	**0.035**	17.46 (8.75)	15.41 (8.05)	0.060
Follow‐up time, months, median (IQR)		545	34.4 (22.5– 35.3)	34.5 (21–35.3)	34.4 (23–35.3)	0.81	34.4 (21.2–35.2)	34.4 (22.5–35.3)	0.67

HADS, Hospital Anxiety and Depression Scale.

* Includes irritable bowel syndrome, fibromyalgia, chronic fatigue syndrome, temporomandibular disorder, and migraines.

^†^ Medications and other treatments were categorised into groups by likely mechanism of action and type: 0 = none, 1 = peripheral action, 2 = central action, and 3 = opioids.

Bold values statistically significant at *P* < 0.05.

### Statistical Analyses and Sample Size

Baseline characteristics, PPS and USS scores, change scores, GRA and other QOL assessments over the 3‐year follow‐up were compared among the *K* = 4 functional cluster subgroups using chi‐square tests, anova, or *t*‐tests as appropriate. The PPS and USS score change over the 3‐year follow‐up was calculated as the score at the last non‐missing visit – baseline score (Week 4 in‐clinic visit). Functional clusters were dichotomised as subgroup 0 (moderate symptom score improvement) vs subgroups 1–3 (slight improvement, no change and slight worsening, respectively); analysed separately for PPS and USS as dependent binary outcomes.

The primary longitudinal analysis was designed to identify baseline factors that demonstrated statistically significant association with classification into subgroup 0 trajectories (moderate improver), in contrast to subgroups (1–3) trajectories, after adjustments for run‐in period averages and baseline in‐clinic levels. Using binary logistic regression modelling, a ‘Score Statistic’ for rank ordering a sequence of ‘All Possible Regressions’ among potentially relevant factors was implemented. Following this selection of the ‘best subset of factors’, a logistic regression model was implemented using a stepwise selection procedure with *P* < 0.15 for inclusion and *P* < 0.05 as retention criteria. Initially, only variables with no missing data were considered eligible for inclusion. Then variables with missing data for <3% of the sample (16 patients) were added to the variable selection process, adjusting for baseline and run‐in period averages for PPS or USS scores. Significant variables (*P* < 0.05) were added to the model selected via stepwise selection and retained if significant.

A minority of patients who had previously been enrolled in the MAPP EPS had slightly different inclusion criteria (pain could be rated at ‘0’), a potentially longer disease course, and previous clinical research exposure. As these factors may be associated with different symptom trajectories, we compared trajectory membership for EPS returnees vs others using a chi‐square test. Similarly, cluster membership was also compared between patients whose follow‐up was administratively truncated (due to termination of data collection) vs others who completed the 3‐year study.

The planned sample size for the UCPPS cohort within the SPS was 620 patients, divided equally among men and women, providing adequate power for the overarching SPS aims [3]. Power analyses for the realised sample size of enrolled patients with UCPPS who also completed the baseline in‐clinic visit (Week 4) and had at least one follow‐up visit were not conducted. Statistical analyses were performed using the Statistical Analysis System (SAS) 9.4 (SAS Institute Inc., Cary, NC, USA). Statistical significance was defined as *P* < 0.05, unless otherwise specified.

## Results

A total of 545 patients were included with a mean (sd) age of 45.5 (15.6) years, 65% female gender, average UCPPS symptom duration 12 years, and median (interquartile range [IQR]) follow‐up of 34.4 (22.5–35.3) months. Follow‐up (as expected) was shorter for those who could not complete 3 years of data collection (*n* = 229; median [IQR] 19.9 [11.1–25.5] months) vs the remainder (*n* = 316; median [IQR] 35.2 [34.7–36.2] months). In all, 80 patients were EPS returnees. At least one chronic overlapping pain condition (COPC) was reported by 61%, and widespread pain by 31%. The median (range) number of medications at baseline overall and for UCPPS were seven (0–32) and two (0–13), respectively. Over 80% reported at least one medication for UCPPS at baseline, including an oral opioid for 127 (23.3%). Presence of Hunner's lesions at cystoscopy could be ascertained in only 209 patients based on records review; among those, 28 (13.4%) had Hunner's lesions.

**Fig. 1:**
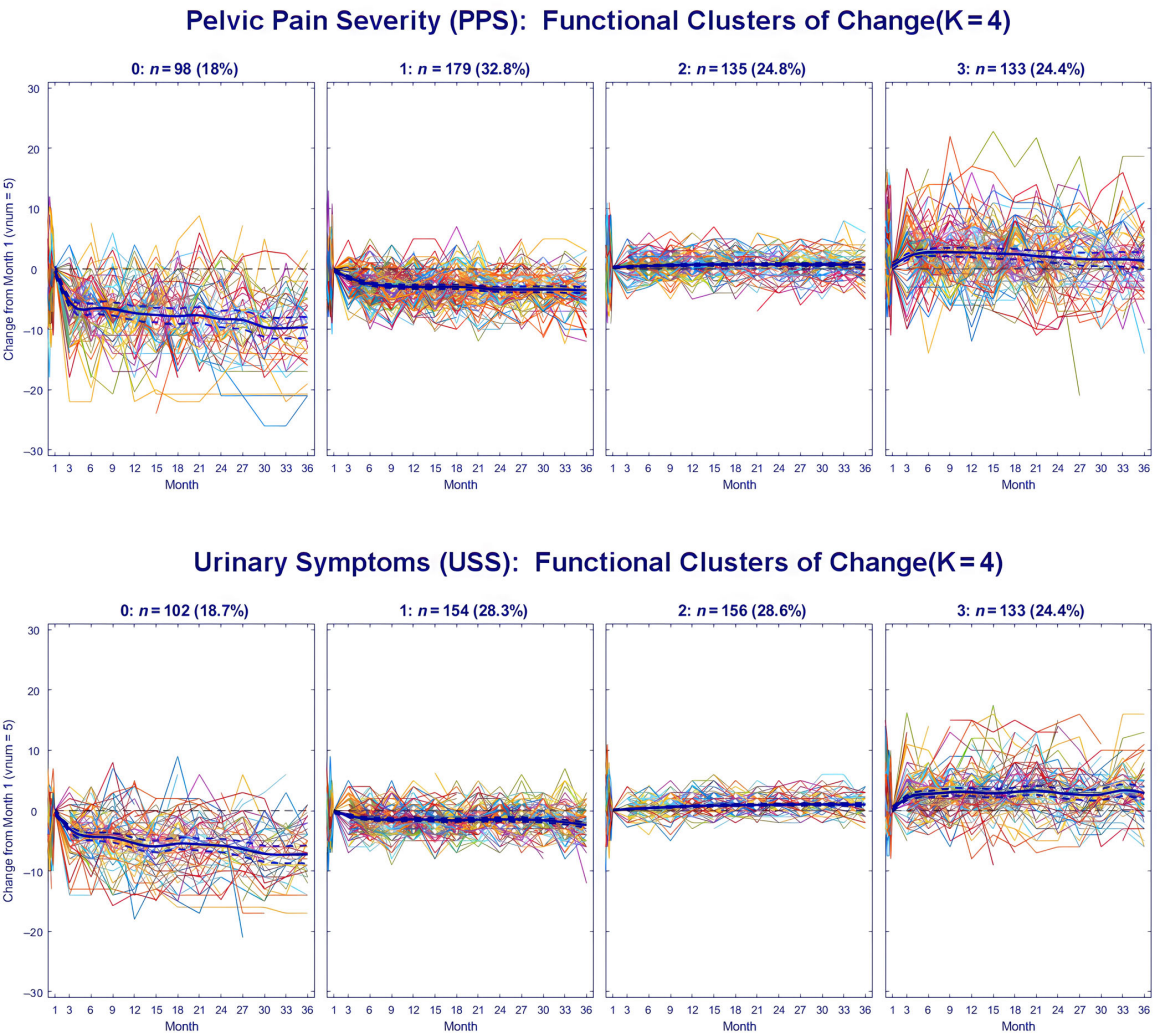
Functional clusters of change (longitudinal trajectories) in PPS and USS over the 3‐year follow‐up (*n* = 545). The *x*‐axis represents months of follow‐up time from enrolment. Score change (*y*‐axis) was calculated as the score at the last non‐missing visit – baseline score (Week 4 visit). Solid lines correspond to ‘Spaghetti plot’ for each patient; dashed lines correspond to ‘Profile plot and 95% confidence limits’ for the mean change of each cluster.

Four significantly different 3‐year symptom trajectory clusters were identified for PPS and USS. The PPS score trajectories (Fig. 1) and score changes (Table S3a) differed across cluster (*P* < 0.001). Based on trajectory shape and PPS score changes, symptom improvement was seen in subgroup 0 (*n* = 98 [18.0%], mean score change −8.5), compared with smaller changes in subgroups 1 (*n* = 179 [32.8%], mean score change −3.2, slight improvement), 2 (*n* = 135 [24.8%], mean score change 0.35, no change), and 3 (*n* = 133 [24.4%], mean score change 1.77, slight worsening). Self‐reported symptom improvement (GRA) was more likely for PPS subgroup 0 with 63% reporting moderate or marked improvement, while many fewer of those in the other groups reported improvement 36%, 23%, and 39% of subgroups 1, 2, and 3, respectively (*P* < 0.001).

Similarly, *K* = 4 USS 3‐year symptom trajectory clusters were identified, which included 19%, 28%, 29% and 24% of patients in subgroups 0, 1, 2, and 3, respectively (Fig. 1, Table S3b). Again, clusters differed by trajectory and mean score change, with group 0 reporting greater improvement (mean score change −6.4; 52% with moderate/marked improvement), with smaller changes in subgroups 1, 2, and 3 (mean score changes −1.9, 0.65, and 2.8; 44%, 28%, and 34% moderate/marked improvement, respectively). There was only moderate correlation between PPS and USS symptom trajectory groups, with ~40% of the PPS improved group also in the USS improved group and vice versa (Table S4). In all, 30% (*n* = 161) were moderately improved in one or both outcomes.

Other patient‐reported outcomes were compared between PPS and USS trajectory subgroups. Changes in all outcomes (GUPI QOL, SF‐12 Physical and Mental Components, PROMIS Sleep Disturbance and Fatigue) were better (improved) in subgroup 0 PPS and USS when compared to the other subgroups. Trends were significant across all clusters for each outcome (*P* < 0.01 for all; Fig. S1).

**Fig. 2:**
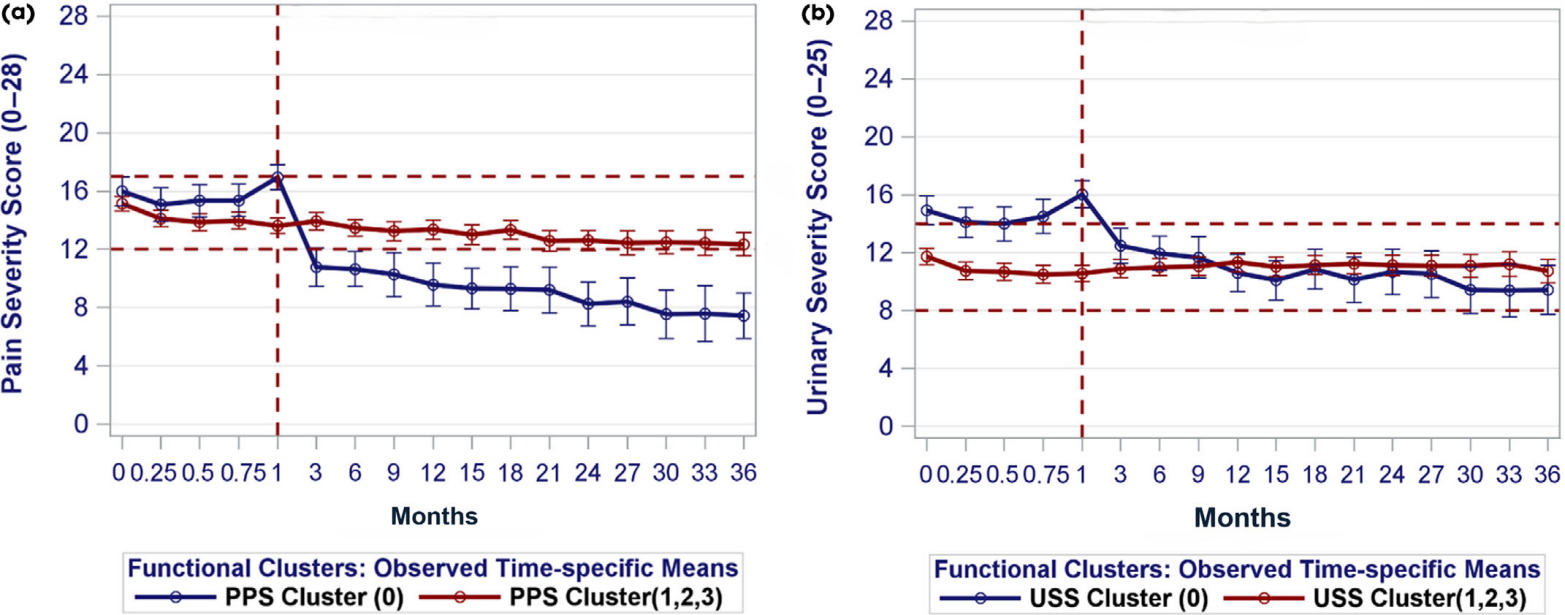
Change in (**a**) PPS and (**b**) USS scores over the 3‐year follow‐up for cluster 0 (moderately improved) vs 1–3 (all others). The *x*‐axis depicts months of follow‐up since enrolment. Observed time‐specific mean scores (*y*‐axis) are plotted (circles) with 95% CIs (error bars). The vertical dashed line marks the baseline visit (at Week 4 or 1 month). The horizontal lines indicate score tertiles at baseline (1 month).

In remaining analyses, the moderately improved subgroup (0) was compared to the other subgroups (1–3, not improved). Longitudinally, the largest change in PPS in subgroup 0 was seen in the first 3 months of follow‐up, after the run‐in period, with more gradual improvement over the remaining 33 months (Fig. 2). The USS improved subgroup pattern was similar, although with less change overall. For both outcomes, subgroups 1–3 had minimal to no change in these outcomes over 3 years.

Baseline demographic and clinical characteristics overall, and in PPS and USS cluster 0 compared to clusters 1–3, are presented in Table 2. Patients with PPS improvement compared to non‐improvers were more likely to be female, to report a lower maximum UCPPS pain medication category, to report having a flare at baseline, and to have higher perceived stress. Patients who improved in USS scores compared to non‐improvers were younger, more often female and were more likely to report a CPOC, a flare at baseline, a flare within the past 3 months, fatigue, and anxiety.

The final multivariable model for PPS improvement (cluster 0) over 3 years included baseline and run‐in period average PPS scores. Patients with greater PPS score at baseline were more likely to improve, whereas a high run‐in average PPS score (suggesting consistently higher pain over the run‐in period) was associated with less likelihood of improvement (Table 3a). The adjusted effect of patients reporting a flare at baseline was attenuated (*P* = 0.25) by the much stronger effects of baseline and run‐in period average PPS scores (*P* < 0.001) in the final PPS model. Demographic and clinical factors associated with pain improvement were also identified, including the type of pain medication used, female sex, sleep dysfunction, and level of education. Patients who reported a higher ‘category’ of medication type for UCPPS (particularly opioid use), male sex, higher education level, and higher baseline sleep disturbance were less likely to have long‐term PPS improvement. Greater lower back and genital pain and more urinary urgency in the run‐in period also were associated with lower odds of improvement, while higher average pain rating during the run‐in period had a borderline association with improvement (*P* = 0.05).

**Table 3a:** Baseline predictors of improvement (membership in cluster 0 vs clusters 1–3) in pain and urinary symptoms over the 3‐year follow‐up: PPS.

Predictor	OR (95% CI)	*P*
Baseline PPS score (0–28)*	1.49 (1.43–1.66)	<0.001
Run‐in average PPS score (0–28)*	0.75 (0.66–0.85)	<0.001
UCPPS medication category (0–3)^†^		
0: None	Reference	
1: Peripheral	0.64 (0.32–1.29)	0.20
2: Central	0.42 (0.20–0.90)	0.025
3: Opioids	0.21 (0.09–0.48)	<0.001
Female gender	2.21 (1.22–4.13)	0.010
Low back pain on body map (yes/no; run‐in average)	0.43 (0.21–0.86)	0.021
PROMIS sleep disturbance score	0.97 (0.84–0.995)	0.023
Education (0: < college, 1: college degree, 2: grad/prof degree)	0.67 (0.46–0.95)	0.028
Genital pain on body map (0–4; run‐in average)^‡^	0.73 (0.55–0.97)	0.033
ICSI item 1 ‘strong need to urinate’ (0–5; run‐in average)^§^	0.81 (0.65–0.99)	0.043
Average GUPI pain (0–10; run‐in average)^¶^	1.25 (1.00–1.57)	0.048

Estimates obtained using multiple logistic regression. Final model included all variables listed and 537 patients.

* Baseline PPS score and the run‐in period average PPS score were included in the model to adjust for regression to the mean.

^†^ Medications for UCPPS were categorised into groups by likely mechanism of action and type: 0: none, 1: peripheral action, 2: central action, and 3: opioids (see Table 1 itemisation).

^‡^ Number of genital areas with pain present on the genital body map (0–4).

^§^ ICSI Item 1: During the past month, how often have you felt the strong need to urinate with little or no warning? 0 (not at all), 1 (less than one time in five), 2 (less than half the time), 3 (about half the time), 4 (more than half the time), 5 (almost always).

^¶^ GUPI item 4: Which number best describes your AVERAGE pain or discomfort on the days that you had it, over the last week? Numerical rating scale: 0 (no pain) to 10 (pain as bad as you can imagine).

Similarly, the baseline factors associated with USS improvement (cluster 0) vs all others (clusters 1–3) included the baseline and run‐in average USS scores (with similar directional associations as for PPS; Table 3b). The adjusted effect of patients reporting a flare at baseline was attenuated (*P* = 0.77) by the much stronger effects of baseline and run‐in period average USS scores (*P* < 0.001) in the final USS model. In addition, patients’ characteristics associated with USS improvement included age (older patients less likely to improve), recent cystoscopic treatment for UCPPS (more likely to improve), and baseline UCPPS treatment (patients who did not report any current UCPPS treatments more likely to improve). Other associated factors included the run‐in averages of urinary frequency (higher frequencies more likely to improve) and non‐urological pain (higher severity less likely to improve).

**Table 3b:** Baseline predictors of improvement (membership in cluster 0 vs clusters 1–3) in pain and urinary symptoms over the 3‐year follow‐up: USS.

Predictor	OR (95% CI)	*P*
Baseline USS score (0–25)*	1.83 (1.59–2.15)	<0.001
Run‐in average USS score (0–25)*	0.56 (0.47–0.67)	<0.001
Age (years)	0.97 (0.96–0.99)	0.002
Cystoscopic treatment for UCPPS within past month of baseline	6.86 (1.83–25.40)	0.004
Any UCPPS treatment (medicine or non‐medicine) vs no treatment	0.27 (0.09–0.78)	0.013
ICSI item 2 urinary frequency (run‐in average)^†^	1.70 (1.12–2.59)	0.013
Non‐PPS (0–10; run‐in average)^‡^	0.89 (0.80–0.99)	0.038

Estimates obtained using multiple logistic regression. Final model included all variables listed and 543 patients.

* Baseline USS score and the run‐in period average USS score were included in the model to adjust for regression to the mean.

^†^ ICSI item 2: During the past month, have you had to urinate less than 2 h after you finished urinating? 0 (not at all), 1 (less than one time in five), 2 (less than half the time), 3 (about half the time), 4 (more than half the time), 5 (almost always).

^‡^ ‘Please rate the overall severity of any persistent pain symptoms that were NOT UROLOGIC OR PELVIC PAIN SYMPTOMS (e.g., back pain, headache, etc.) over the past 2 weeks: Rate from 0 (no symptoms) to 10 (symptoms as bad as they can be)’.

Subgroup analyses did not differ significantly from overall results (data not shown). First, functional cluster membership was not different among patients who previously enrolled in the MAPP EPS compared to other enrollees. Second, patients whose follow‐up ended early due to study closure were not different in cluster membership from those who completed follow‐up.

## Discussion

Over the 3‐year follow‐up, we identified four trajectories for patients’ pain and urinary UCPPS symptoms (moderate improvement, slight improvement, no change, and slight worsening). Symptom trajectory groups correlated in expected directions with QOL and other assessments. Moderate improvers (18–20% of patients for both pelvic pain and urinary symptoms, 30% for at least one outcome) had significant improvements in other QOL outcomes, were more likely to report improvement in GRA, and their average PPS and USS score changes exceeded what has been identified as clinically meaningful change [15]. Moderate improvers also had higher baseline scores and were more likely to report having a flare at baseline, characterised as ‘pain higher than usual’. Baseline factors associated with improvement in pain and urinary symptoms, after adjusting for baseline and run‐in period symptom scores, included clinical factors, such as treatments. Patients reporting oral opioid use at baseline had lower odds of meaningful improvement in their pain score after follow‐up and those who underwent cystoscopic treatment for their UCPPS (i.e., fulguration of Hunner's ulcer or hydrodistension) had greater odds of improvement in urinary symptoms.

A limited literature has studied UCPPS symptom natural history. Our results are similar to Naliboff et al. [1], who found 22% and 21% of patients improved over 1 year in pain and urinary symptoms, respectively. That MAPP study used more frequent, biweekly assessments with a similar functional clustering technique. A subset of those patients had extended follow‐up for a median (range) of 4.6 (1.3–9.0) years, and those who improved in the initial year generally maintained that improvement over the next 2–9 years [2]. Others have reported higher rates of long‐term UCPPS improvement. One study in women with IC/BPS found 35% reported improvement at a median of 33 months [17], and a retrospective clinic‐based study of women with IC/BPS reported 59% symptom improvement after mean follow‐up of 16.6 years [18]. Differences in study populations, symptom duration, and disease acuity may explain these different outcomes.

Few predictors of long‐term UCPPS symptom trajectories have been reported. In the EPS 1‐year study, only physical health‐related QOL and baseline severity were predictors of pain and urinary symptom patterns [1]. Poorer sleep was associated with lack of improvement in univariable analyses, but not when adjusted for other variables, including baseline severity. In the SPS, poor sleep was associated with less improvement in pain over the 3‐year follow‐up, even after adjusting for baseline severity. This may be a baseline marker for more severe symptoms or a marker of a specific UCPPS phenotype that is less likely to improve.

After adjusting for pain severity at baseline and during the run‐in period, UCPPS medication category was the strongest predictor of pain outcomes in this study. Compared to those reporting no UCPPS medications at baseline, the 23% of patients reporting opioid use were less likely to improve (odds ratio [OR] 0.21; *P* < 0.001). Those reporting centrally active medications (e.g., amitriptyline) were also less likely to improve (OR 0.42; *P* = 0.025), whereas patients reporting peripherally active medications were non‐significantly less likely to improve (OR 0.64; *P* = 0.20). Again, these results may serve as markers of severity, but medication type may also indicate varied phenotypes of UCPPS. Patients prescribed centrally active medications and opioids may be more likely to have widespread pain and non‐pelvic pain syndromes, suggesting a central vs local disease mechanism.

Recent treatment via cystoscopy was associated with a greater likelihood of USS improvement during follow‐up (OR 6.86; *P* = 0.004). Our interpretation of this is limited by lack of information about the specific treatment performed and the small number of patients reporting this treatment at baseline (3.5%). However, cystoscopy with treatment of Hunner's ulcers is known to be effective. Our result supports the recommendation for earlier diagnosis and treatment of Hunner's lesions as in the 2022 AUA IC/BPS guideline [19].

The MAPP II SPS was designed with a 4‐week run‐in period, based on findings from MAPP I EPS that suggested important regression to the mean effects. Our SPS analyses confirmed that the average of symptoms assessed over four weekly visits prior to baseline (‘run‐in average’) was a strong independent predictive factor, second only to the baseline measure, in predicting classification into cluster 0 symptom trajectories. In both PPS and USS models, the run‐in average score had the opposite effect as the single baseline score on likelihood of Group 0 membership. For example, if the baseline PPS score was high, but the run‐in average PPS score was much lower, there was a greater chance of regression to the mean (early symptom improvement), so inclusion of both variables in the models may better adjust for this phenomenon. These results overall confirm that one single baseline assessment in UCPPS is not adequate to anchor a longitudinal study, perhaps because of inherent variability in these self‐reported symptom assessments. The use of run‐in averages over four data points also allowed us to adjust for symptom stability, while maximising the number of patients with non‐missing data. Use of run‐in averages might also adjust for substantial within‐day variability that occurs in patients with UCPPS [20].

Study strengths include the prospective, multicentre design and long‐term follow‐up. Also, the 4‐week run‐in period allowed us to better adjust for potential ‘regression to mean’ phenomena than in prior studies. We also acknowledge limitations. We considered pain and urinary symptoms as separate outcomes, even though these symptom patterns are not statistically independent. The observational nature of this research limits conclusions related to predictors of improvement, as treatments were not standardised and likely differed based on baseline factors. Also, results from this research population enrolled from tertiary care settings may not be generalisable to all patients. Finally, in these analyses we considered only baseline predictors of longer‐term symptom patterns. Future research is needed to consider how interval treatment changes or other time‐dependent factors may influence symptom outcomes.

## Conclusions

In a large UCPPS cohort, 30% had symptom improvement in pain and/or urinary symptoms over the 3‐year follow‐up. Baseline factors associated with improvement may represent markers of a less severe or localised phenotype and/or treatment effects. These results provide clinicians with information about UCPPS symptom trajectories and support other MAPP findings of important phenotypic differences in patients with UCPPS.

## Author Contributions

Catherine S. Bradley: conceptualisation, methodology, writing – original draft. Mengying You: methodology, formal analysis, data curation, writing – review and editing. Wensheng Guo: conceptualisation, methodology, writing – review and editing, supervision. Niloofar Afari: conceptualisation, writing – review and editing. Priyanka Gupta: writing – review and editing. Karl J. Kreder: conceptualisation, writing – review and editing, funding acquisition. John N. Krieger: conceptualisation, writing – review and editing. H. Henry Lai: conceptualisation, writing – review and editing, funding acquisition. Susan K. Lutgendorf: conceptualisation, writing – review and editing. Bruce D. Naliboff: conceptualisation, writing – review and editing. Siobhan Sutcliffe: conceptualisation, writing – review and editing. Frank Tu: writing – review and editing. David A. Williams: conceptualisation, writing – review and editing. Tara McWilliams: formal analysis, data curation, writing – review and editing. Larissa Rodriguez: writing – review and editing, funding acquisition. J. Richard Landis: conceptualisation, methodology, formal analysis, writing – review and editing, supervision, funding acquisition.

## Funding Information

Funding for the MAPP Research Network was obtained under a cooperative agreement from National Institute of Diabetes and Digestive and Kidney Diseases (NIDDK), National Institutes of Health (NIH) (DK82370, DK82342, DK82315, DK82344, DK82325, DK82345, DK82333, and DK82316).

## Disclosure of Interests

All authors report NIH support for the research performed. In addition, the following is reported: Bradley: additional NIH‐research funding; Elsevier‐textbook royalties, journal editor stipend and travel support; American Board of Ob‐Gyn‐division member and examiner stipend and travel support; American Urogynecologic Society‐board member travel support. H. Henry Lai: Medtronic‐research grant, Society of Urodynamics, Female Pelvic Medicine and Urogenital Reconstruction‐travel support; AUA‐chair stipend. Frank Tu: NIH‐research grant; Wolters Kluwers‐royalties for UpToDate chapter; International Pelvic Pain Society‐advisory board member, no payments; Maipl‐advisor, stock/stock options. Tara McWilliams: Johnson & Johnson, Bayer‐stock/stock options. Larissa Rodriguez: Elsevier‐royalties; University of Washington‐honoraria visiting professor; US patent‐7531355 B2; Foundation for Women's Health‐advisory board member. J. Richard Landis: University of Washington‐CTSA advisory board member. Mengying You, Wensheng Guo, Niloofar Afari, Priyanka Gupta, Karl J. Kreder, John N. Krieger, Susan K. Lutgendorf, Bruce D. Naliboff, Siobhan Sutcliffe, and David A. Williams: no additional competing interests to declare.

## Prior Presentation

Abstract presented (moderated poster) at the Society of Urodynamics, Female Pelvic Medicine and Urogenital Reconstruction (SUFU) 2025 Winter Meeting, 26 February to 1 March 2025, Rancho Mirage, CA, USA.

## Supporting information


**Table S1.** The MAPP SPS patient assessments.
**Table S2.** Variables tested as potential model covariates for pelvic pain and urinary symptom trajectory models.
**Table S3.** Symptom scores at baseline and the run‐in period, symptom score change and self‐reported improvement status at last non‐missing visit over the 3‐year follow‐up by functional cluster groups in PPS and USS.
**Table S4.** Patients grouped by PPS and USS trajectories.
**Fig. S1.** Association of PPS (a) and USS (b) trajectories with changes in other patient‐reported outcome scores.

**Appendix.** MAPP II Research Network Study Group.

## Data Availability

Data from The MAPP II Study of Urologic Chronic Pelvic Pain Syndrome: The Trans‐MAPP SPS (MAPP II) ([version 1] https://doi.org/10.58020/2gk3‐hy95) reported here are available for request at the NIDDK Central Repository (NIDDK‐CR) website, Resources for Research (R4R), https://repository.niddk.nih.gov/.
