# Supplementary material for: Urologic chronic pelvic pain syndrome 3‐year symptom trajectories: the Multidisciplinary Approach to the Study of Chronic Pelvic Pain (MAPP) Symptom Patterns Study
Source: BJU Int. 2025 Dec 2;137(2):312–22. doi: 10.1111/bju.70087 (PMC12789841; doi:10.1111/bju.70087)
Supplement: Supplementary file 1 — Table S1. The MAPP SPS patient assessments. Table S2. Variables tested as potential model covariates for pelvic pain and urinary symptom trajectory models. Table S3. Symptom scores at baseline and the run‐in period, symptom score change and self‐reported improvement status at last non‐missing visit over the 3‐year follow‐up by functional cluster groups in PPS and USS. Table S4. Patients grouped by PPS and USS trajectories. Fig. S1. Association of PPS (a) and USS (b) trajectories with changes in other patient‐reported outcome scores. [file BJU-137-312-s001.docx]

**Supplemental Material: Contents**

**Trans-MAPP Symptom Patterns Study (SPS) Inclusion and Exclusion Criteria**

**Statistical Supplemental Methods: Functional Clustering**

**Table S1** MAPP SPS Patient Assessments

**Table S2** MAPP SPS variables tested as potential predictors for pelvic pain and urinary symptom trajectory models.

**Table S3** Pelvic Pain Severity and Urinary Symptom Severity scores and change by cluster groups

**Table S4** Participants grouped by Pelvic Pain Severity and Urinary Symptom Severity trajectories

**Figure S1** Association of Pelvic Pain Severity and Urinary Symptom Severity trajectories with change in other patient reported outcomes during follow-up

**Trans-MAPP Symptom Patterns Study (SPS) Inclusion and Exclusion Criteria**

Eligibility criteria for the SPS protocol are nearly identical to those for the MAPP I Epidemiology and Phenotyping Study (EPS) by design.

**Inclusion criteria**

UCPPS participants:

(a) UCPPS symptoms present for a majority of the time during most recent 3 months,

(b) age 18 years and above, and

(c) response 1 or greater on the bladder/prostate or pelvic pain/pressure/discomfort

scale during past 2 weeks*

**Exclusion criteria**

- symptomatic urethral stricture

- neurological disease or disorder affecting the bladder

- bladder fistula

- history of cystitis caused by tuberculosis, radiation therapy or chemotherapy

- prior augmentation cystoplasty or cystectomy

- active autoimmune or infectious disorder

- history of pelvic cancer

- current major psychiatric disorder

- severe cardiac, pulmonary, renal, or hepatic disease

- unilateral orchalgia (without pelvic symptoms)

- prior prostate procedures (transurethral microwave thermotherapy, transurethral needle ablation, balloon dilation, prostate cryosurgery, or laser procedure)

* Re‐enrolling MAPP I participants with a pain/pressure/discomfort score of 0 were also eligible for SPS to provide insight into factors influencing UCPPS symptom resolution. However, only 3 of 80 EPS returnees had PPS = 0 at enrollment (0.6% of all participants).

**Statistical Supplemental Methods: Functional Clustering**

Owing to the extensive subject heterogeneity and differential UCPPS symptom variability over time, we adopted a functional clustering approach to generate K longitudinal trajectory subgroups for each of the two primary UCPPS symptom severity scores (PPS, USS). Using PPS and USS scores from every visit, each participant was classified iteratively into a “best fitting” trajectory subgroup for each of K = 2, …, 8 clusters. The statistical methods for this approach were utilized in prior MAPP UCPPS data applications (1, 2). Recently, the algorithm was refined to increase computational efficiency and validated extensively within a simulation study (3).

To enable the calculation of within-subject longitudinal change, missing values at the baseline anchor time point (in-clinic at 4 weeks) were imputed using a single imputation procedure based on a linear mixed-effects model. In this model, time (measured in months) was modeled as a continuous predictor using cubic polynomial terms to flexibly capture nonlinear trajectories. The model included fixed effects for linear, quadratic, and cubic time terms to characterize the overall population trend, and random effects for the intercept, linear, quadratic, and cubic terms to account for subject-specific deviations. Model estimation was performed using maximum likelihood, and predicted values at the baseline time point were used as imputed values, leveraging each subject’s observed outcome data from neighboring time points.

For both PPS and USS, the functional clustering algorithm was implemented in MATLAB (MathWorks, 2021a; Natick, MA) to generate K=2, …, 8 subgroups, clustering on the individual longitudinal change scores, for which negative values represent severity decline and positive values represent increased severity. This approach also allows covariate-adjusted models to predict functional cluster membership for each participant, as well as includes adjustment for the anchor baseline level. The optimal number of clusters was also computed separately for PPS and USS based on an information criterion (3). For each of PPS and USS, this iterative functional cluster procedure finished in approximately 20 min for our real data applications on a personal computer desktop (Processor: Inter Core i7-9700, CPU 3.00GHz, RAM: 16GB).

1. Naliboff BD, Stephens AJ, Lai HH, et al. Clinical and Psychosocial Predictors of Urological Chronic Pelvic Pain Symptom Change in 1 Year: A Prospective Study from the MAPP Research Network. *J Urol*. Oct 2017;198(4):848-857. doi:10.1016/j.juro.2017.05.065
2. Stephens-Shields AJ, Clemens JQ, Jemielita T, et al. Symptom Variability and Early Symptom Regression in the MAPP Study: A Prospective Study of Urological Chronic Pelvic Pain Syndrome. *J Urol*. Nov 2016;196(5):1450-1455. doi:10.1016/j.juro.2016.04.070
3. Guo W, You M, Yi J, Pontari MA, Landis JR. Functional mixed effects clustering with application to longitudinal urologic chronic pelvic pain syndrome symptom data. *J Am Stat Assoc*. 2022;117(540):1631-1641. doi:10.1080/01621459.2022.2066536

**Table S1**

Patient-reported assessments/questionnaires included in the MAPP SPS and considered as potential baseline predictors in this study.

| **Domain** | **Measure** | **Measure: additional information** | **Reference** |
| --- | --- | --- | --- |
| **Genito-urinary** | Genitourinary Pain Index (GUPI) | UCPPS symptoms and quality of life | Clemens JQ, Calhoun EA, Litwin MS, et al. Validation of a modified National Institutes of Health chronic prostatitis symptom index to assess genitourinary pain in both men and women. *Urology*. Nov 2009;74(5):983-7 |
|  | Interstitial Cystitis Symptom Index (ICSI) | Interstitial cystitis symptom frequency | O'Leary MP, Sant GR, Fowler FJ, Jr., Whitmore KE, Spolarich-Kroll J. The interstitial cystitis symptom index and problem index. *Urology*. May 1997;49(5A Suppl):58-63 |
|  | RAND Interstitial Cystitis Epidemiology (RICE) questionnaire |  | Berry SH, Bogart LM, Pham C, et al. Development, validation and testing of an epidemiological case definition of interstitial cystitis/painful bladder syndrome. *J Urol*. May 2010;183(5):1848-52 |
|  | AUA Symptom Index | Urinary symptoms and quality of life | Barry MJ, Fowler FJ, O’Leary MP et al: The American Urological Association symptom index for benign prostatic hyperplasia. The Measurement Committee of the American Urological Association. J Urol 1992; 148: 1549 |
|  |  |  |  |
| **Pain** | Brief Pain Inventory |  | Cleeland C. *The Brief Pain Inventory: User Guide*. MD Anderson Cancer Center; 2009 |
|  | MAPP II body map | Modified from the Collaborative Health Outcomes Information Registry body map; pain rated 0-10 in 76 sites grouped in 13 regions | Clemens JQ, Locke K, Jr., Landis JR, et al. Validation of a simple body map to measure widespread pain in urologic chronic pelvic pain syndrome: A MAPP Research Network study. *Neurourol Urodyn*. Mar 2024;43(3):727-37 |
|  | painDETECT | Neuropathic pain symptoms | Freynhagen R, Baron R, Gockel U, Tölle TR. painDETECT: a new screening questionnaire to identify neuropathic components in patients with back pain. *Curr Med Res Opin*. Oct 2006;22(10):1911-20 |
|  |  |  |  |
| **Non-urologic symptoms/conditions** | Complex Medical Symptom Inventory (CMSI) |  | Williams DA, Schilling S. Advances in the assessment of fibromyalgia. *Rheum Dis Clin North Am*. May 2009;35(2):339-57 |
|  | Patient Reported Outcomes Measurement Information System (PROMIS) Fatigue, PROMIS Sleep Disturbance | Fatigue and sleep disturbance | Cella D, Riley W, Stone A, et al. The Patient-Reported Outcomes Measurement Information System (PROMIS) developed and tested its first wave of adult self-reported health outcome item banks: 2005-2008. *J Clin Epidemiol*. Nov 2010;63(11):1179-94 |
|  | International Physical Activity Questionnaire (IPAQ) | Physical activity | Craig, C. L., et al. (2003). "International physical activity questionnaire: 12-country reliability and validity." Med Sci Sports Exerc **35**(8): 1381-95 |
|  | Multiple Ability Self-Report Questionnaire (MASQ) | Cognitive dysfunction | Seidenberg, M., et al. (1994). "Development and validation of a Multiple Ability Self-Report Questionnaire." J Clin Exp Neuropsychol **16**(1): 93-104 |
|  | Ten-Item Personality Inventory (TIPI) | Personality dimensions | Goldberg, L. R., et al. (2006). "The international personality item pool and the future of public-domain personality measures." J Res Pers **40**(1): 84-96 |
| **Quality of life/general health** | SF-12 | Generic physical and mental health-related quality of life | Ware J, Jr., Kosinski M, Keller SD. A 12-Item Short-Form Health Survey: construction of scales and preliminary tests of reliability and validity. *Med Care*. Mar 1996;34(3):220-33 |
|  | World Health Organization Disability and Assessment Schedule (WHO-DAS) | Overall health and disability | Pösl, M., et al. (2007). "Psychometric properties of the WHODASII in rehabilitation patients." Qual Life Res 16(9): 1521-31 |
|  |  |  |  |
| **Mental health, psychosocial** | Hospital Anxiety and Depression Scale (HADS) | Anxiety, depression | Zigmond AS, Snaith RP. The hospital anxiety and depression scale. *Acta Psychiatr Scand*. Jun 1983;67(6):361-70 |
|  | Perceived Stress Scale | Stress | Cohen S, Kamarck T, Mermelstein R. A global measure of perceived stress. *J Health Soc Behav*. Dec 1983;24(4):385-96 |
|  | Positive and Negative Affect Scale (PANAS) | Affect | Watson D, Clark LA, Tellegen A. Development and validation of brief measures of positive and negative affect: the PANAS scales. *J Pers Soc Psychol*. Jun 1988;54(6):1063-70 |
|  | Coping Strategies Questionnaire (CSQ) – Catastrophizing | Catastrophizing | Keefe FJ, Brown GK, Wallston KA, Caldwell DS. Coping with rheumatoid arthritis pain: catastrophizing as a maladaptive strategy. *Pain*. Apr 1989;37(1):51-6 |
|  | Childhood Traumatic Events Scale (CTES) and Recent Traumatic Events Scale (RTES) | Life adversity – childhood and adult | Pennebaker JW, Susman JR. Disclosure of traumas and psychosomatic processes. *Soc Sci Med*. 1988;26(3):327-32 |

**Table S2.** Variables tested as potential model covariates for pelvic pain and urinary symptom trajectory models. Variables collected at baseline or during the run-in period were considered candidates if missing data occurred in less than 3% of the sample.

| **Variable(s)** | **Variable Type** | **Visit Collected** | | |
| --- | --- | --- | --- | --- |
|  |  | **Screening (wk 0)** | **Run-in visits (wks 0, 1, 2, 3)** | **Baseline (wk 4)** |
| Age | Demographic | X |  |  |
| Black race | Demographic | X |  |  |
| Gender | Demographic | X |  |  |
| Education | Demographic | X |  |  |
| Living with partner | Demographic | X |  |  |
| Clinical diagnosis of chronic prostatitis only (no to IC/BPS, males only) | Medical History | X |  |  |
| Total number of medications (both overall and for UCPPS considered) | Medications |  |  | X |
| Maximum medication category (both overall and for UCPPS considered, 0-3) | Medications |  |  | X |
| Individual medications used for UCPPS within last month (all listed in Table 1 considered individually) | Medications |  |  | X |
| Individual non-medicine treatments used for UCPPS within last month (all listed in Table 1 considered individually) | Treatment |  |  | X |
| Maximum treatment category (both overall and for UCPPS considered, 0-3) | Medications/ Treatment |  |  | X |
| Pelvic floor muscle tenderness (# areas in examination with tenderness, 0-6, females only) | Examination |  |  | X |
| # areas with pain (any (y/n) and areas with pain intensity >=4 considered) | Body Map |  | X |  |
| Pain in any areas of specific non-pelvic regions (front head, back head, low back, upper back all considered individually) | Body Map |  | X |  |
| Pain in pelvic areas (any (y/n), pain intensity >=4 (y/n), and # areas with pain considered) | Body Map |  | X |  |
| Average pain intensity for pelvic areas with pain | Body Map |  | X | X |
| Number of genital areas with pain | Genital Body Map |  | X | X |
| Urinary Symptom Severity score | PRO |  | X | X |
| Pelvic Pain Severity score | PRO |  | X | X |
| GUPI (individual items 1-6 and score) | PRO |  | X | X |
| ICSI (individual items 1-4 and score) | PRO |  | X | X |
| RICE (painful filling, painful urgency, subtype) | PRO |  | X | X |
| RICE Bladder Symptom Impact score | PRO |  |  | X |
| AUASI total score | PRO |  |  | X |
| BPI Item 6 | PRO |  | X | X |
| BPI Pain Interference Score | PRO |  |  | X |
| Pain Detect Neuropathic Pain score | PRO |  |  | X |
| # symptoms (both non-pelvic and urinary considered, CMSI) | PRO |  |  | X |
| Pelvic/bladder discomfort (y/n, CSMI) | PRO |  |  | X |
| CMSI scores (both Somatic Awareness and Sensory Sensitivity) | PRO |  |  | X |
| Fibromyalgia scores (Symptom Severity, Widespread Pain, and total all considered) | PRO |  | X | X |
| IPAQ Most strenuous level of activity | PRO |  |  | X |
| PROMIS Fatigue T score | PRO |  |  | X |
| PROMIS Sleep Disturbance T score | PRO |  |  | X |
| SF-12 (Mental Health, Physical Health Scores both considered) | PRO |  |  | X |
| Rating of non-urologic pain symptoms (0-10) | PRO |  | X | X |
| Rating of mood overall (0-10) | PRO |  |  | X |
| GRA Item 1: Spend rest of life with symptoms like today | PRO |  |  | X |
| GRA Item 2: Current symptoms compared to start of study | PRO |  |  | X |

IC/BPS, interstitial cystitis/bladder pain syndrome; UCPPS, urologic chronic pelvic pain syndrome; CMSI, Complex Medical Symptoms Inventory; GUPI, Genitourinary Pain Index; ICSI, Interstitial Cystitis Symptom Index; RICE, RAND Interstitial Cystitis Epidemiology questionnaire; AUASI, American Urological Association Symptom Index; BPI, Brief Pain Inventory; PROMIS, Patient Reported Outcomes Measurement Information System; GRA, Global Response Assessment.

**Table S3.** Symptom scores at baseline and the run-in period, symptom score change and self-reported improvement status at last non-missing visit over 36-months follow-up by functional cluster groups in Pelvic Pain Severity and Urinary Symptom Severity.

**2a. Pelvic Pain Severity (PPS)**

| **Outcome** | **Overall N=545** | **PPS: 0 N=98** | **PPS: 1 N=179** | **PPS: 2 N=135** | **PPS: 3 N=133** | **P-Value*** |
| --- | --- | --- | --- | --- | --- | --- |
| PPS score, baseline | 14.20 (5.62) | 16.93 (4.25) | 14.99 (5.28) | 14.63 (5.76) | 10.68 (5.13) | <0.001 |
| PPS score, run-in average** | 14.56 (5.12) | 15.41 (4.61) | 14.36 (4.92) | 15.59 (5.30) | 13.13 (5.25) | 0.067 |
| PPS score change*** | -2.05 (5.56) | -8.47 (5.33) | -3.19 (3.32) | 0.35 (2.36) | 1.77 (5.75) | <0.001 |
| GRA: at least moderately improved**** (n (%); n=530) | 205 (38.68%) | 60 (63.16%) | 64 (36.36%) | 31 (23.48%) | 50 (39.37%) | <0.001 |
| **2b. Urinary Symptom Severity (USS)** | | | | | |  |
| **Outcome** | **Overall N=545** | **USS: 0 N=102** | **USS: 1 N=154** | **USS: 2 N=156** | **USS: 3 N=133** | **P-Value*** |
| USS score, baseline | 11.59 (6.20) | 16.02 (4.78) | 12.58 (5.39) | 9.99 (6.96) | 8.92 (4.89) | <0.001 |
| USS score, run-in average** | 11.60 (5.85) | 14.44 (5.01) | 11.95 (5.52) | 10.38 (6.76) | 10.45 (4.85) | <0.001 |
| USS score change*** | -0.88 (4.52) | -6.44 (4.17) | -1.92 (2.64) | 0.65 (1.90) | 2.80 (4.14) | <0.001 |
| GRA: at least moderately improved****  (n (%); n=530) | 205 (38.68%) | 51 (52.04%) | 67 (44.37%) | 43 (28.48%) | 44 (33.85%) | 0.003 |
|  |  |  |  |  |  |  |

The Global Responses Assessment (GRA) queried, “As compared to when you started the study, how would you rate your overall symptoms now?” with 1:“markedly worse,” 2:“moderately worse,” 3:“slightly worse,” 4:“no change,” 5:“slightly improved,” 6:“moderately improved,” or 7:“markedly improved” as responses.

Data presented as mean (SD) unless otherwise indicated.

*p-value was calculated using t test for group 0 vs. groups 1-3 combined.

**Score average across 4 weekly assessments (weeks 0-3)

***Score change = score at last non-missing visit - baseline (week 4) score

**** GRA score = (6 or 7) at last non-missing visit

**Table S4:** Participants grouped by Pelvic Pain Severity (PPS) and Urinary Symptom Severity (USS) trajectories. There was only moderate correlation between PPS and USS symptom trajectory groups, with 40% (39 of 98) of the PPS improved group (group 0) also included in the USS improved group (group 0). Similarly, 38% (39 of 102) of the USS improved group also were included in the PPS improved group.

| **36-Month Symptom Trajectory** | **USS Cluster** | | | |  |
| --- | --- | --- | --- | --- | --- |
|  | **0 (N=102)** | **1 (N=154)** | **2 (N=156)** | **3 (N=133)** | **Overall (N=545)** |
| **PPS Cluster** |  |  |  |  |  |
| **0 (N=98)** | 39  39.8%  38.2% | 30  30.6%  19.5% | 15  15.3%  9.6% | 14  14.3%  10.5% | 98  100%  18.0% |
| **1 (N=179)** | 32  17.9%  31.4% | 61  34.1%  39.6% | 52  29.1%  33.3% | 34  19.0%  25.6% | 179  100%  32.8% |
| **2 (N=135)** | 18  13.3%  17.6% | 35  25.9%  22.7% | 53  39.3%  34.0% | 29  21.5%  21.8% | 135  100%  24.8% |
| **3 (N=133)** | 13  9.8%  12.7% | 28  21.1%  18.2% | 36  27.1%  23.1% | 56  42.1%  42.1% | 133  100%  24.4% |
| **Overall** | 102  18.7%  100% | 154  8.3%  100% | 156  28.6%  100% | 133  24.4%  100% | 545  100%  100% |

Data presented as N, row %, and column %.

**Figure S1.** Association of PPS (a) and USS (b) trajectories with changes in other patient-reported outcome scores

PPS, Pelvic Pain Severity; USS, Urinary Symptom Severity; GUPI, Genitourinary Pain Index; PROMIS, Patient Reported Outcomes Measurement Information System.

**S1a.**

**S1b.**
