# Supplementary material for: Urologic chronic pelvic pain syndrome 3‐year symptom trajectories: the Multidisciplinary Approach to the Study of Chronic Pelvic Pain (MAPP) Symptom Patterns Study
Source: BJU Int. 2025 Dec 2;137(2):312–22. doi: 10.1111/bju.70087 (PMC12789841; doi:10.1111/bju.70087)
Supplement: Supplementary file 2 — Appendix. MAPP II Research Network Study Group. [file BJU-137-312-s002.pdf]

MAPP II Research Network Study Group

University of Pennsylvania Data Coordinating Core

J. Richard Landis, PhD, Core Dir.
Dina Appleby, MS
Ted Barrell, BA
Ro-Pauline Doe, BA
John T. Farrar, MD, MSCE, PhD
Melissa Fernando, MPH
Laura Gallagher, MPH, CCRP
Xiaoling Hou, MS
Tamara Howard, MPH
Thomas Jemielita, MS
Natalie Kuzla, MA
Kenneth Locke, Jr.
Robert M. Moldwin, MD
Craig Newcomb, MS
Michel A. Pontari, MD
Nancy Robinson-Garvin, PhD
Sandra Smith, AS
Alisa Stephens-Shields, PhD
Sally Thompson, MS
Yanli Wang, MS
Xingmei Wang, MS

University of Colorado Denver Tissue Analysis & Technology Core

M. Scott Lucia, MD, Core Dir.
Adrie van Bokhoven, PhD, Co-Dir.
Robert Dayton, Jr
Oluwatosin Ibrahim, MS
Andrea A. Osypuk, BS
Hannah Schumann, BS
Chelsea S. Triolo, BS
R. Storey Wilson, MS

National Institutes of Diabetes & Digestive and Kidney Diseases

Chris Mullins, PhD
Tamara G. Bavendam, MD
Ziya Kirkali, MD
John W. Kusek, PhD

MAPP Network Executive Committee

J. Quentin Clemens, MD, FACS, MSci, Network Chair, 2013-
J. Richard Landis, PhD
M. Scott Lucia, MD
Robert M. Moldwin, MD
Michel A. Pontari, MD
Chris Mullins, PhD
Tamara Bavendam, MD
Ziya Kirkali, MD

DISCOVERY SITES

Northwestern University

David J. Klumpp, PhD, Co-Dir.
Anthony J. Schaeffer, MD, Co-Dir.
Christina Arroyo
Michael Bass, PhD
Sarah C Flury, MD
James W. Griffith, PhD
Charles J. Heckman II, PhD
Mingchen Jiang, PhD
Laurie Keefer, PhD
Robert Lloyd, PhD
Darlene S. Marko, RN, BSN, CCRC
Richard Miller, PhD
Todd Parrish, PhD
Frank Tu, MD, MPH
Ryan Yaggie

University of California, Los Angeles / University of Southern California & The PAIN Neuroimaging Core

Emeran A. Mayer, MD, Co-Dir.
Larissa V. Rodríguez, MD, Co-Dir.
Jeffry Alger, PhD
Cody P. Ashe-McNalley
Ben Ellingson, PhD
Nuwanthi Heendeniya
Lisa Kilpatrick, PhD
Cara Kulbacki
Jason Kutch, PhD
Jennifer S. Labus, PhD
Bruce D. Naliboff, PhD
Suzanne R. Smith, RN, NP
Jeff Loh-Doyle, MD

University of Iowa

Karl J. Kreder, MD, MBA, Dir.
Catherine S. Bradley, MD, MSCE
Mary Eno, RN, RA
Isabelle Hardy, MBA
Susan K. Lutgendorf, PhD
Michael A. O'Donnell, MD
Stacy McMichael, BS
Vince Magnotta, PhD
Brad Erickson, MD

University of Michigan

Daniel J. Clauw, MD, Co-Dir.; Network Chair, 2008-2013
J. Quentin Clemens, MD, FACS, MSci, Co-Dir.; Network Chair, 2013-
Suzie As-Sanie, MD
Priyanka Gupta, MD
Megan E. Halvorson, BS, CCRP
Richard E. Harris, PhD
Steven E. Harte, PhD
Eric Ichesco, BS
Susanne Quallich, PhD, NP
Katherine A. Scott, RN, BSN
Andrew Schrepf, PhD
David A. Williams, PhD

Washington State University / University of Washington

Dedra Buchwald, MD, WSU Dir.
Eric Strachan, PhD, UW Dir.
Niloofar Afari, PhD, UCSD
Tamara Bacus, BS
John Krieger, MD
Kenneth Maravilla, MD
Jane Miller, MD
Jean Kallhoff, ARNP
Cara Kulbaki, BS
Swati Rane, PhD
Stephanie Richey, BS
Kelly Robertson, BS
Susan O. Ross, RN, MN
Suzette Sutherland, MD
Claire C. Yang, MD

Washington University in St. Louis

Gerald L. Andriole, MD, Co-Dir..
H. Henry Lai, MD, Co-Dir.
Robert W. Gereau IV, PhD,
Siobhan Sutcliffe, PhD, ScM, MHS
Graham A. Colditz, MD, DrPH
Simon Haroutounian, PhD
Jeffrey P. Henderson, MD, PhD
Theresa M. Spitznagle, PT, DPT, WCS
Barry A. Hong, PhD
Akinkunle Owoso, MD
Timothy J. Ness, MD, PhD
Georg Deutsch, PhD
Aleksandra P. Klim, RN, MHS, CCRC
Vivien C. Gardner, RN, BSN
Deborah Ksiazek, RN, BSN
Patricia Hayden, RN, BSN
Susan Mueller, RN, BSN
Maria Payne, BA

NON-RECRUITING DISCOVERY SITES

Cedars-Sinai Medical Center

Jennifer Anger, MD, MPH
James Ackerman, MA
A. Lenore Ackerman, MD, PhD
Jeena Cha, BS, CCRP
Karyn Eilber, MD
Michael Freeman, PhD
Jayoung Kim, PhD
Jennifer Van Eyk, PhD
Wei Yang, PhD

Queens University

J. Curtis Nickel, MD, FRCSC, Dir.
Garth D. Ehrlich, PhD, [Drexel COM]

Harvard Medical School/ Boston Children's Hospital

Marsha A. Moses, PhD, Dir.
Andrew C. Briscoe
David Briscoe, MD
Adam Curatolo, BA
John Froehlich, PhD
Richard S. Lee, MD
Monisha Sachdev, BS
Keith R. Solomon, PhD
Hanno Steen, PhD
